# Crystal structure and absolute configuration of (3a*S*,4*S*,5*R*,7a*R*)-2,2,7-trimethyl-3a,4,5,7a-tetra­hydro-1,3-benzodioxole-4,5-diol

**DOI:** 10.1107/S2056989015014590

**Published:** 2015-08-06

**Authors:** Mario A. Macías, Leopoldo Suescun, Enrique Pandolfi, Valeria Schapiro, Gaurao D. Tibhe, Álvaro W. Mombrú

**Affiliations:** a Cryssmat-Lab/Cátedra de Física/DETEMA, Facultad de Química, Universidad de la República, Montevideo, Uruguay; b Grupo INTERFASE, Universidad Industrial de Santander, Carrera 27, Calle 9, Ciudad Universitaria, Bucaramanga, Colombia; c Departamento de Química Orgánica, Facultad de Química, Universidad de la República, Montevideo, Uruguay

**Keywords:** crystal structure, ep­oxy­cyclo­hexenones, absolute configuration, chiral crystal, chemoenzymatic strategy

## Abstract

The absolute configuration of the title compound, C_10_H_16_O_4_, determined as 3a*S*,4*S*,5*R*,7a*R* on the basis of the synthetic pathway, was confirmed by X-ray diffraction. The mol­ecule contains a five- and a six-membered ring that adopt twisted and envelope conformations, respectively. The dihedral angle between the mean planes of the rings is 76.80 (11)° as a result of their *cis*-fusion. In the crystal, mol­ecules are linked by two pairs of O—H⋯O hydrogen bonds, forming chains along [010]. These chains are further connected by weaker C—H⋯O inter­actions along [100], creating (001) sheets that inter­act only by weak van der Waals forces.

## Abstract 2

The crystal structure of enanti­opure (3a*S*,4*S*,5*R*,7a*R*)-2,2,7-trimethyl-3a,4,5,7a- tetra­hydro-1,3-benzodioxole-4,5-diol shows that the absolute configuration determined from the synthesis pathway agrees with that determined by X-ray analysis.

## Chemical context   

**Figure 1:**
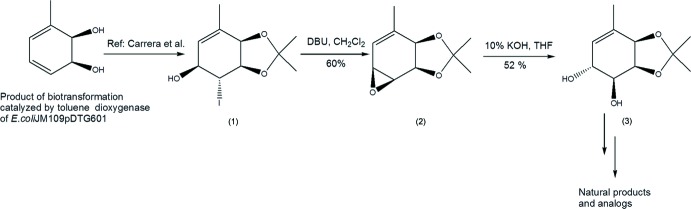
Synthesis pathway and structural scheme of the chiral C_10_H_16_O_4_ compound.

Compounds containing an ep­oxy­cyclo­hexenone skeleton are very inter­esting, not only because of their wide spectrum of biological activities, but also because of their synthetically challenging chemical structures (Pandolfi *et al.*, 2013 ▸). A biotransformation of toluene leads to a chiral diol (see Fig. 1 ▸) which is used as a precursor in enanti­oselective syntheses of ep­oxy­cyclo­hexenone compounds. Model compounds of the central core of ambuic acid (Labora *et al.*, 2008 ▸), (+)- and (−)-bromoxone (Labora *et al.*, 2010 ▸), an ep­oxy­quinol analog (Heguaburu *et al.*, 2010 ▸), gabosine A, ent-epoformin and ent-epiepoformin (Labora *et al.*, 2011 ▸) have been prepared starting from the same precursor. The title compound, diol (3) (see Fig. 1 ▸) has been prepared from iodo­hydrin (1), which, as indicated earlier, can be easily synthesized *via* biotransformation of toluene (Carrera *et al.*, 2007 ▸).
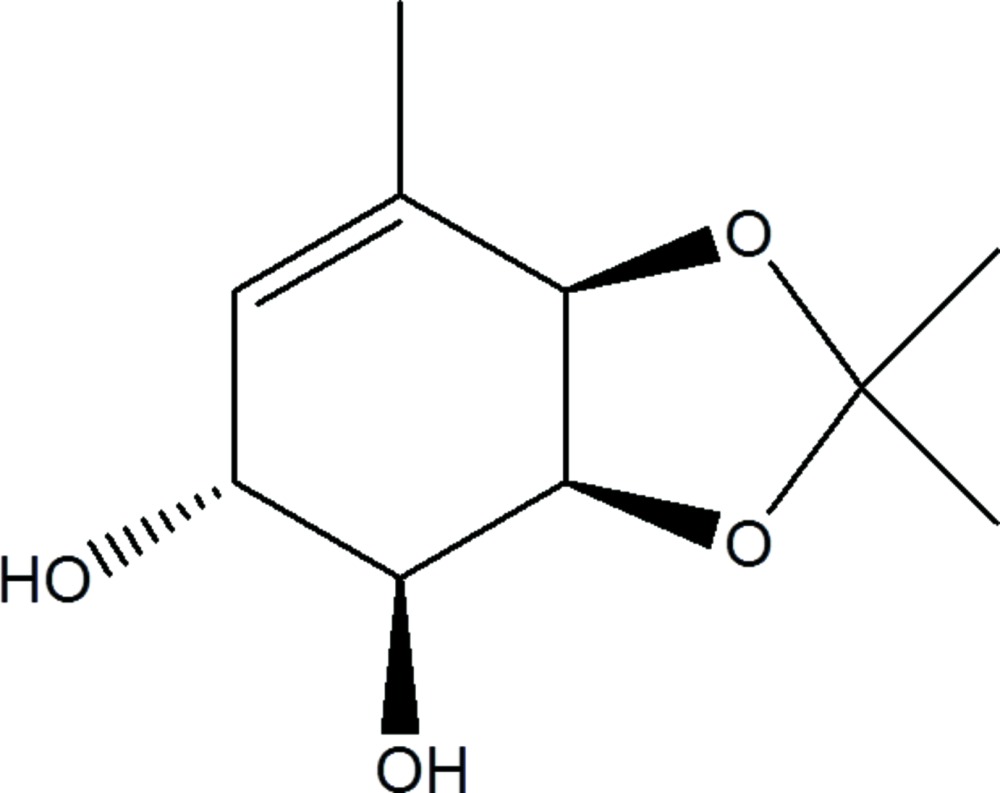


## Structural commentary   

**Figure 2:**
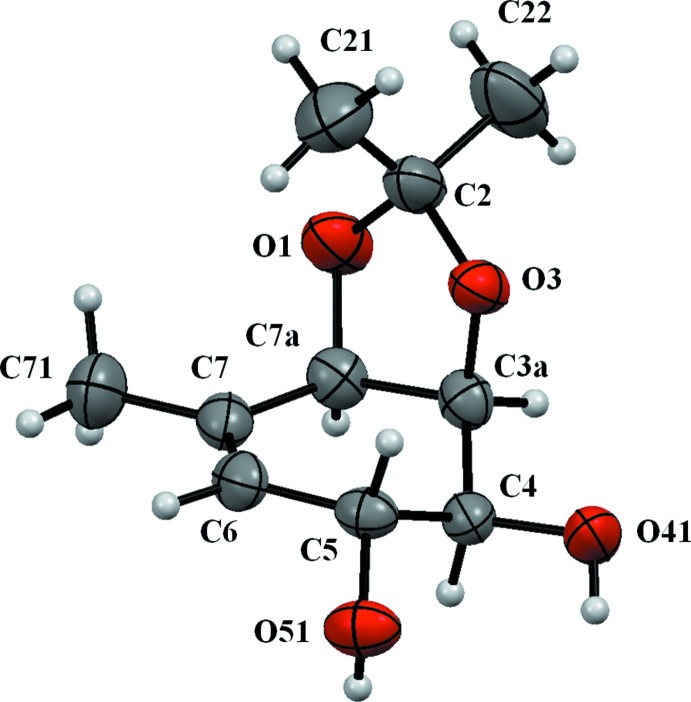
The mol­ecular structure of the title compound, showing the anisotropic displacement ellipsoids drawn at the 50% probability level.

Fig. 2 ▸ shows the mol­ecule of the title compound. The absolute configuration of the title compound, determined as 3a*S*,4*S*,5*R*,7a*R* on the basis of the synthetic pathway, was confirmed by X-ray diffraction on the basis of anomalous dispersion of light atoms only. The five-membered ring (O1–C2–O3–C3A–C7A) adopts a twisted conformation with puckering parameters *Q*(2) = 0.342 (2) Å and φ = 122.1 (3)°. The six-membered ring (C3A–C4–C5–C6–C7–C7A) adopts an envelope conformation with atom C4 as the flap. In this case, the puckering parameters are *Q* = 0.466 (2) Å, θ = 52.1 (2) and φ = 50.8 (3)°. The fused rings are nearly perpendicular with a dihedral angle of 76.20 (11)° as a result of their *cis*-fusion.

## Supra­molecular features   

**Figure 3:**
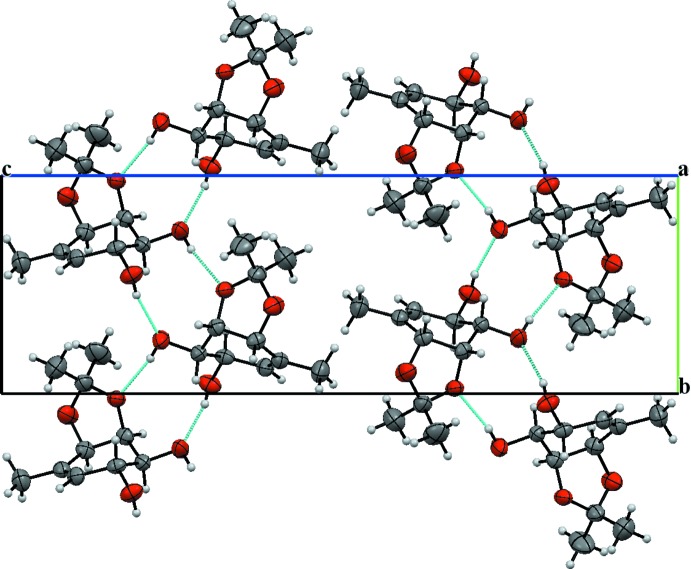
Packing of the title compound, viewed along [100], showing hydrogen-bonded chains of mol­ecules

In the crystal structure, the mol­ecules are connected in the three crystallographic directions by inter­molecular inter­actions of different strengths (Table 1 ▸). In the [010] direction hydrogen bonds O41—H41⋯O3^i^ and O51—H51⋯O41^i^ [symmetry code: (i) −*x*, *y* + 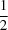, −*z* + 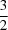] join mol­ecules into chains that are further connected by weaker C7A—H7A⋯O51^ii^ [symmetry code: (ii) *x* + 1, *y*, *z*] hydrogen bonds along [100], forming (001) sheets. Hydrogen bonds of the O—H⋯O type generate 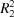
(10) motifs (Fig. 3 ▸). There are only weak van der Waals forces acting between neighbouring (001) sheets.

## Database survey   

A search of the Cambridge Structural Database (CSD Version 5.36 with one update; Groom & Allen, 2014 ▸) of the 3a,4,5,7a-tetra­hydro-1,3-benzodioxole skeleton gave 30 hits, of which only 20 had no additional fused rings. In all cases, the six-membered ring displays an envelope conformation with atom C4 as a flap. The orientation of the flap with respect to the plane of the envelope can be determined from the C7—C7A—C3A—C4 or the H7A—C7A—C3A—H3A torsion angles (with very similar values due to the geometry of the *cis*-fused rings). The C7—C7A—C3A—C4 torsion angle is positive if the flap atom is located on the opposite side of the plane (defined by the remaining five atoms of the cyclo­hexene ring) to O1 and O3 of the 1,3-dioxole ring, as observed in the title compound [33.5 (2)°]. 12 of the 20 mentioned structures, show a positive torsion angle with minimum and maximum values of 17.2 and 36.4°, respectively. From analysis of the above-mentioned torsion angle and the equatorial/axial orientation of the C4 and C5 substituents in the 20 structures, there is no clear trend that allows the relative orientation of the flap to be predicted based only on the size or kind of the substituents.

## Synthesis and crystallization   

The synthesis of the title compound was carried out through the inter­mediate epoxide (2) (see Fig. 1 ▸). Iodo­hydrin (1) (0.6 mmol, 0.18 g) was dissolved in dry di­chloro­methane (5 mL) and 1,8-di­aza­bicyclo[5.4.0]undec-7-en (DBU) (0.8 mmol, 0.12 g) was added at room temperature. The reaction was stirred for 24 h After completion of the reaction, the mixture was diluted with saturated NH_4_Cl solution (20 mL) and extracted with di­chloro­methane (3 x 10 mL). The combined organic layers were washed with saturated NaCl solution (10 mL), dried (Na_2_SO_4_) and filtered. Concentration of the filtrate, followed by flash chromatography (hexa­nes:ethyl acetate 93:7) yielded (2) (0.063 g, 60%). FT–IR (KBr): 2983, 2926, 2856, 1672, 1371. 1H-NMR (400 MHz, CDCl_3_) δ: 6.01 (*m*, 1H), 4.40 (*m*, 2H), 3.58 (*m*, 1H), 3.42 (*t*, *J* = 4.0 Hz, 1H), 1.91 (*s*, 3H), 1.53 (*s*, 3H), 1.41 (*s*, 1H). For the synthesis of diol (3), epoxide (2) (0.27 mmol, 0.05 g) was dissolved in THF (25 mL) and 10% KOH (aq., 25 mL) was added. This mixture was refluxed for 4 h. After completion of the reaction, the mixture was diluted with di­chloro­methane (20 mL) and the organic phase was washed with 10% HCl until neutralization, washed with saturated NaCl solution (10 mL), dried with (Na_2_SO_4_) and filtered. Concentration of the filtrate, followed by flash chromatography (ethyl acetate:hexa­nes 4:6) yielded (3) (0.02g, 52%). Crystals suitable for X-ray structure analysis were obtained by dissolving (3) in the minimum volume of ethyl acetate, adding hexa­nes until the solution became slightly turbid and slowly evaporating the solvent at room temperature. (m.p. = 385–386 K). FT–IR (KBr): 3402, 1637, 1371. ^1^H NMR (400 MHz, CDCl3) δ: 5.45 (*s*, 1H), 4.48 (*m*, 2H), 4.33 (*m*, 1H), 3.59 (*m*, 1H), 2.52 (*bs*, 1H), 2.30 (*bs*, 1H), 1.79 (*s*, 3H), 1.38 (*s*, 3H), 1.35 (*s*, 3H).

## Refinement details   

Crystal data, data collection and structure refinement details are summarized in Table 2 ▸. H atoms bonded to C were placed in calculated positions (C—H = 0.95–1.00 Å) and included as riding contributions with isotropic displacement parameters set to 1.2–1.5 times of the *U*_eq_ of the parent atom. H atoms belonging to OH groups were located in ΔF maps and freely refined.

## Supplementary Material

Crystal structure: contains datablock(s) I, New_Global_Publ_Block. DOI: 10.1107/S2056989015014590/gk2644sup1.cif

Structure factors: contains datablock(s) I. DOI: 10.1107/S2056989015014590/gk2644Isup2.hkl

Supporting information file. DOI: 10.1107/S2056989015014590/gk2644Isup3.cml

CCDC reference: 1416687

Additional supporting information:  crystallographic information; 3D view; checkCIF report

## Figures and Tables

**Table 1:** Hydrogen-bond geometry (, )

*D*H*A*	*D*H	H*A*	*D* *A*	*D*H*A*
O41H41O3^i^	0.82(3)	2.04(3)	2.849(2)	171(3)
O51H51O41^i^	0.80(3)	2.04(3)	2.826(2)	167(2)
C7AH7AO51^ii^	0.98	2.44	3.299(3)	146

Symmetry codes: (i) 
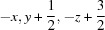
; (ii) 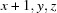.

**Table 2:** Experimental details

Crystal data	
Chemical formula	C_10_H_16_O_4_
*M*_r_	200.23
Crystal system, space group	Orthorhombic, *P*2_1_2_1_2_1_
Temperature (K)	293
*a*, *b*, *c* ()	6.1230(13), 7.5163(17), 23.347(5)
*V* (^3^)	1074.5(4)
*Z*	4
Radiation type	Cu *K*
(mm^1^)	0.79
Crystal size (mm)	0.28 0.18 0.14

Data collection	
Diffractometer	Bruker D8 Venture/Photon 100 CMOS
Absorption correction	Multi-scan (*SADABS*; Bruker, 2013 ▸)
*T*_min_, *T*_max_	0.643, 0.752
No. of measured, independent and observed [*I* > 2(*I*)] reflections	29451, 1967, 1951
*R*_int_	0.030
(sin /)_max_ (^1^)	0.603

Refinement	
*R*[*F* ^2^ > 2(*F* ^2^)], *wR*(*F* ^2^), *S*	0.029, 0.078, 1.18
No. of reflections	1967
No. of parameters	139
H-atom treatment	H atoms treated by a mixture of independent and constrained refinement
_max_, _min_ (e ^3^)	0.14, 0.11
Absolute structure	Flack *x* determined using 782 quotients [(*I* ^+^)(*I* )]/[(*I* ^+^)+(*I* )] (Parsons *et al.*, 2013 ▸)
Absolute structure parameter	0.01(3)

Computer programs: *APEX2* and *SAINT* (Bruker, 2013 ▸), *SHELXS97* (Sheldrick, 2008 ▸), *SHELXL2014* (Sheldrick, 2015 ▸) and *Mercury* (Macrae *et al.*, 2008 ▸).

## supplementary crystallographic information

### Crystal data

**Table:**

C_10_H_16_O_4_	*F*(000) = 432
*M**_r_* = 200.23	*D*_x_ = 1.238 Mg m^−^^3^
Orthorhombic, *P*2_1_2_1_2_1_	Cu *K*α radiation, λ = 1.54178 Å
Hall symbol: P 2ac 2ab	θ = 3.8–66.7°
*a* = 6.1230 (13) Å	µ = 0.79 mm^−^^1^
*b* = 7.5163 (17) Å	*T* = 293 K
*c* = 23.347 (5) Å	Parallelepiped, colorless
*V* = 1074.5 (4) Å^3^	0.28 × 0.18 × 0.14 mm
*Z* = 4	

### Data collection

**Table:**

Bruker D8 Venture/Photon 100 CMOS diffractometer	1967 independent reflections
Radiation source: Cu Incoatec microsource	1951 reflections with *I* > 2σ(*I*)
Helios X-ray optical focusing and monochromatization module	*R*_int_ = 0.030
Detector resolution: 10.4167 pixels mm^-1^	θ_max_ = 68.4°, θ_min_ = 3.8°
π and ω scans	*h* = −7→6
Absorption correction: multi-scan (*SADABS*; Bruker, 2013)	*k* = −9→9
*T*_min_ = 0.643, *T*_max_ = 0.752	*l* = −28→28
29451 measured reflections	

### Refinement

**Table:**

Refinement on *F*^2^	Hydrogen site location: mixed
Least-squares matrix: full	H atoms treated by a mixture of independent and constrained refinement
*R*[*F*^2^ > 2σ(*F*^2^)] = 0.029	*w* = 1/[σ^2^(*F*_o_^2^) + (0.0278*P*)^2^ + 0.2133*P*] where *P* = (*F*_o_^2^ + 2*F*_c_^2^)/3
*wR*(*F*^2^) = 0.078	(Δ/σ)_max_ < 0.001
*S* = 1.18	Δρ_max_ = 0.14 e Å^−^^3^
1967 reflections	Δρ_min_ = −0.11 e Å^−^^3^
139 parameters	Extinction correction: *SHELXL2014* (Sheldrick, 2015), Fc^*^=kFc[1+0.001xFc^2^λ^3^/sin(2θ)]^-1/4^
0 restraints	Extinction coefficient: 0.0063 (9)
Primary atom site location: structure-invariant direct methods	Absolute structure: Flack *x* determined using 782 quotients [(*I*^+^)-(*I*^-^)]/[(*I*^+^)+(*I*^-^)] (Parsons *et al.*, 2013)
Secondary atom site location: difference Fourier map	Absolute structure parameter: 0.01 (3)

### Special details

**Table:**

Geometry. All e.s.d.'s (except the e.s.d. in the dihedral angle between two l.s. planes) are estimated using the full covariance matrix. The cell e.s.d.'s are taken into account individually in the estimation of e.s.d.'s in distances, angles and torsion angles; correlations between e.s.d.'s in cell parameters are only used when they are defined by crystal symmetry. An approximate (isotropic) treatment of cell e.s.d.'s is used for estimating e.s.d.'s involving l.s. planes.

### Fractional atomic coordinates and isotropic or equivalent isotropic displacement parameters (Å^2^)

**Table:**

	*x*	*y*	*z*	*U*_iso_*/*U*_eq_	
O3	0.1718 (2)	0.02424 (17)	0.83031 (6)	0.0431 (4)	
C3A	0.2609 (3)	0.1964 (3)	0.81761 (8)	0.0379 (4)	
H3A	0.3868	0.1850	0.7919	0.045*	
O1	0.4078 (3)	0.0973 (2)	0.90222 (7)	0.0562 (5)	
C7A	0.3342 (3)	0.2591 (3)	0.87622 (8)	0.0405 (5)	
H7A	0.4583	0.3403	0.8719	0.049*	
H41	−0.026 (5)	0.337 (4)	0.7201 (12)	0.066 (9)*	
H51	−0.197 (5)	0.545 (4)	0.8006 (10)	0.051 (8)*	
O41	0.0349 (3)	0.2538 (2)	0.73585 (6)	0.0499 (4)	
O51	−0.2609 (3)	0.4552 (2)	0.80767 (7)	0.0488 (4)	
C7	0.1596 (4)	0.3484 (3)	0.91126 (8)	0.0403 (5)	
C6	−0.0366 (3)	0.3793 (3)	0.88973 (8)	0.0415 (5)	
H6	−0.1387	0.4344	0.9133	0.050*	
C23	0.4527 (6)	−0.1837 (4)	0.85765 (13)	0.0778 (9)	
H23A	0.5376	−0.2248	0.8896	0.117*	
H23B	0.5475	−0.1307	0.8297	0.117*	
H23C	0.3769	−0.2824	0.8407	0.117*	
C2	0.2898 (4)	−0.0479 (3)	0.87792 (9)	0.0484 (5)	
C4	0.0914 (3)	0.3159 (3)	0.79142 (8)	0.0358 (4)	
H4	0.1557	0.4347	0.7873	0.043*	
C5	−0.1064 (3)	0.3320 (2)	0.83012 (9)	0.0370 (4)	
H5	−0.1771	0.2152	0.8317	0.044*	
C71	0.2246 (5)	0.3979 (4)	0.97104 (10)	0.0621 (7)	
H71A	0.1042	0.4554	0.9898	0.093*	
H71B	0.3471	0.4775	0.9698	0.093*	
H71C	0.2639	0.2925	0.9919	0.093*	
C22	0.1246 (6)	−0.1199 (4)	0.91968 (12)	0.0762 (9)	
H22A	0.1985	−0.1646	0.9530	0.114*	
H22B	0.0437	−0.2145	0.9019	0.114*	
H22C	0.0263	−0.0266	0.9308	0.114*	

### Atomic displacement parameters (Å^2^)

**Table:**

	*U*^11^	*U*^22^	*U*^33^	*U*^12^	*U*^13^	*U*^23^
O3	0.0525 (8)	0.0324 (7)	0.0444 (8)	0.0017 (6)	−0.0105 (7)	−0.0019 (6)
C3A	0.0346 (9)	0.0391 (10)	0.0400 (10)	0.0008 (8)	0.0044 (8)	−0.0027 (8)
O1	0.0621 (10)	0.0478 (9)	0.0587 (9)	0.0118 (8)	−0.0243 (8)	−0.0051 (7)
C7A	0.0353 (9)	0.0407 (10)	0.0455 (10)	−0.0020 (9)	−0.0032 (9)	−0.0026 (9)
O41	0.0668 (11)	0.0458 (8)	0.0372 (7)	0.0157 (8)	−0.0080 (7)	−0.0020 (7)
O51	0.0369 (8)	0.0452 (9)	0.0643 (10)	0.0056 (7)	0.0024 (7)	0.0124 (7)
C7	0.0482 (12)	0.0349 (10)	0.0377 (10)	−0.0064 (9)	0.0036 (9)	−0.0010 (8)
C6	0.0428 (11)	0.0414 (11)	0.0405 (10)	0.0022 (9)	0.0124 (9)	0.0014 (9)
C23	0.083 (2)	0.0651 (17)	0.0857 (19)	0.0354 (16)	−0.0191 (16)	−0.0160 (15)
C2	0.0605 (14)	0.0390 (11)	0.0456 (11)	0.0103 (10)	−0.0116 (10)	−0.0022 (9)
C4	0.0408 (10)	0.0323 (9)	0.0343 (9)	0.0000 (8)	0.0034 (8)	−0.0004 (7)
C5	0.0323 (9)	0.0323 (9)	0.0465 (10)	0.0003 (8)	0.0031 (8)	0.0047 (8)
C71	0.0779 (17)	0.0649 (15)	0.0436 (12)	0.0032 (15)	−0.0050 (12)	−0.0076 (11)
C22	0.102 (2)	0.0665 (17)	0.0599 (15)	−0.0030 (18)	0.0010 (15)	0.0110 (13)

### Geometric parameters (Å, º)

**Table:**

O3—C2	1.433 (2)	C6—H6	0.9300
O3—C3A	1.435 (2)	C23—C2	1.503 (3)
C3A—C4	1.503 (3)	C23—H23A	0.9600
C3A—C7A	1.515 (3)	C23—H23B	0.9600
C3A—H3A	0.9800	C23—H23C	0.9600
O1—C2	1.427 (3)	C2—C22	1.505 (4)
O1—C7A	1.432 (3)	C4—C5	1.516 (3)
C7A—C7	1.504 (3)	C4—H4	0.9800
C7A—H7A	0.9800	C5—H5	0.9800
O41—C4	1.421 (2)	C71—H71A	0.9600
O41—H41	0.82 (3)	C71—H71B	0.9600
O51—C5	1.424 (2)	C71—H71C	0.9600
O51—H51	0.80 (3)	C22—H22A	0.9600
C7—C6	1.323 (3)	C22—H22B	0.9600
C7—C71	1.498 (3)	C22—H22C	0.9600
C6—C5	1.499 (3)		
			
C2—O3—C3A	108.04 (16)	O1—C2—C23	108.0 (2)
O3—C3A—C4	111.11 (16)	O3—C2—C23	110.34 (19)
O3—C3A—C7A	101.91 (15)	O1—C2—C22	111.0 (2)
C4—C3A—C7A	112.71 (16)	O3—C2—C22	107.4 (2)
O3—C3A—H3A	110.3	C23—C2—C22	113.9 (2)
C4—C3A—H3A	110.3	O41—C4—C3A	110.06 (15)
C7A—C3A—H3A	110.3	O41—C4—C5	112.08 (17)
C2—O1—C7A	108.78 (15)	C3A—C4—C5	110.92 (15)
O1—C7A—C7	111.85 (17)	O41—C4—H4	107.9
O1—C7A—C3A	102.24 (16)	C3A—C4—H4	107.9
C7—C7A—C3A	114.80 (16)	C5—C4—H4	107.9
O1—C7A—H7A	109.2	O51—C5—C6	112.15 (17)
C7—C7A—H7A	109.2	O51—C5—C4	111.30 (16)
C3A—C7A—H7A	109.2	C6—C5—C4	110.18 (16)
C4—O41—H41	105.6 (19)	O51—C5—H5	107.7
C5—O51—H51	107.4 (19)	C6—C5—H5	107.7
C6—C7—C71	123.5 (2)	C4—C5—H5	107.7
C6—C7—C7A	121.16 (18)	C7—C71—H71A	109.5
C71—C7—C7A	115.4 (2)	C7—C71—H71B	109.5
C7—C6—C5	124.75 (18)	H71A—C71—H71B	109.5
C7—C6—H6	117.6	C7—C71—H71C	109.5
C5—C6—H6	117.6	H71A—C71—H71C	109.5
C2—C23—H23A	109.5	H71B—C71—H71C	109.5
C2—C23—H23B	109.5	C2—C22—H22A	109.5
H23A—C23—H23B	109.5	C2—C22—H22B	109.5
C2—C23—H23C	109.5	H22A—C22—H22B	109.5
H23A—C23—H23C	109.5	C2—C22—H22C	109.5
H23B—C23—H23C	109.5	H22A—C22—H22C	109.5
O1—C2—O3	105.92 (16)	H22B—C22—H22C	109.5
			
C2—O3—C3A—C4	−151.43 (16)	C7A—O1—C2—C23	127.8 (2)
C2—O3—C3A—C7A	−31.14 (19)	C7A—O1—C2—C22	−106.6 (2)
C2—O1—C7A—C7	95.1 (2)	C3A—O3—C2—O1	14.5 (2)
C2—O1—C7A—C3A	−28.2 (2)	C3A—O3—C2—C23	−102.1 (2)
O3—C3A—C7A—O1	35.64 (18)	C3A—O3—C2—C22	133.2 (2)
C4—C3A—C7A—O1	154.80 (17)	O3—C3A—C4—O41	−68.3 (2)
O3—C3A—C7A—C7	−85.7 (2)	C7A—C3A—C4—O41	178.01 (16)
C4—C3A—C7A—C7	33.5 (2)	O3—C3A—C4—C5	56.3 (2)
O1—C7A—C7—C6	−120.5 (2)	C7A—C3A—C4—C5	−57.4 (2)
C3A—C7A—C7—C6	−4.6 (3)	C7—C6—C5—O51	−147.6 (2)
O1—C7A—C7—C71	59.4 (2)	C7—C6—C5—C4	−23.0 (3)
C3A—C7A—C7—C71	175.29 (19)	O41—C4—C5—O51	−60.8 (2)
C71—C7—C6—C5	179.8 (2)	C3A—C4—C5—O51	175.69 (16)
C7A—C7—C6—C5	−0.3 (3)	O41—C4—C5—C6	174.10 (15)
C7A—O1—C2—O3	9.6 (2)	C3A—C4—C5—C6	50.6 (2)

### Hydrogen-bond geometry (Å, º)

**Table:**

*D*—H···*A*	*D*—H	H···*A*	*D*···*A*	*D*—H···*A*
O41—H41···O3^i^	0.82 (3)	2.04 (3)	2.849 (2)	171 (3)
O51—H51···O41^i^	0.80 (3)	2.04 (3)	2.826 (2)	167 (2)
C7*A*—H7*A*···O51^ii^	0.98	2.44	3.299 (3)	146

Symmetry codes: (i) −*x*, *y*+1/2, −*z*+3/2; (ii) *x*+1, *y*, *z*.
